# Emergency department eCPR: an exploratory observational study of human factors

**DOI:** 10.1186/s12873-026-01650-4

**Published:** 2026-06-17

**Authors:** Andrew R. Coggins, Jeremiah E. Cho, Henry Crayton, Amelia Scott, Natalie Kruit, Matthew Oliver

**Affiliations:** 1 The University of Sydney, Block K, Level 6, Westmead Hospital, Sydney, NSW Australia; 2 Department of Emergency Medicine, Block K, Level 6, Westmead Hospital, Sydney, NSW Australia; 3 Department of Anaesthetics, Block K, Level 6, Westmead Hospital, Sydney, NSW Australia; 4 Department of Emergency Medicine, Royal Prince Alfred Hospital, Sydney, NSW Australia; 5 Green Light Institute for Emergency Care, Royal Prince Alfred Hospital, Sydney, NSW Australia

**Keywords:** Extracorporeal cardiopulmonary resuscitation (eCPR), Extracorporeal membrane oxygenation (ECMO), Human factors, Teamwork, Non-technical skills

## Abstract

**Background:**

Extracorporeal cardiopulmonary resuscitation (eCPR) is a low-frequency, high-acuity intervention requiring technical skills and coordinated team performance. This exploratory study observed eCPR cases for elements of teamwork, provider stress, and environmental factors in the Emergency Department (ED). Reid’s conceptual framework domains for human factors in healthcare teams were used as an analytical lens for interpreting the data.

**Methods:**

An exploratory two-phase study was conducted across two eCPR hospital sites over 32 months, with an initial simulation and non-eCPR observation phase used to refine study methods. A subsequent eCPR case phase used convenience sampling of adult cardiac arrests to observe actual eCPR events. Following consent, eCPR team members allocated to key roles (such as team leadership and cannulation) were observed in detail during each case. Patient outcomes included Utstein measures and eCPR timings. Provider outcomes included mobile heart rate monitoring, salivary cortisol, and validated psychometric measures. Teamwork was assessed using the Mayo High-Performance Team Score (MHPTS). Environmental outcomes included measurements of ambient noise levels, team headcounts, and observations of eCPR workflow.

**Results:**

Eighteen providers were observed during six eCPR events. The mean case age was 44.7 years and most patients presented with a shockable rhythm. System features consistently observed in the eCPR cases included predefined roles, individuals assigned to crowd control, and team leader summaries. Causal interpretations are limited by the small sample size and risk of observational bias. Within these limitations, individual provider salivary cortisol measurements were higher after eCPR copared with paired baseline samples. Teamwork scores (MHPTS) were in a moderate to high range (*median = 12; IQR 10–13*) and environmental observations reflected noisy ED spaces and crowding (*headcount median = 20; IQR 18–29*).

**Conclusion:**

This exploratory study found that human factors outcomes can be feasibly assessed during ED eCPR. Predefined roles and structured communication were observed and warrant further study.

**Supplementary Information:**

The online version contains supplementary material available at 10.1186/s12873-026-01650-4.

## Background

Extracorporeal cardiopulmonary resuscitation (eCPR) is an emerging intervention in Emergency Departments (EDs) for highly-selected cases [1–3]. Evidence of benefit in out-of-hospital cardiac arrest (OHCA) is growing, leading more hospitals to develop eCPR systems [4–6]. Extracorporeal membrane oxygenation (ECMO) is challenging to deliver because high-level technical and non-technical skills are required [27]. eCPR is recognised as technically difficult but also relies on a rapid interdisciplinary team response. Therefore, eCPR cases share characteristics with ‘major trauma’ responses in the ED setting. eCPR and trauma calls are typically unscheduled and time-dependent with various important roles taken at short notice by team members that may not have worked together. In contrast to trauma responses, eCPR calls occur less frequently and therefore pose the additional challenge of being high-acuity, low-occurrence (HALO) events [7, 8]. Other eCPR challenges include limited preparation time, limited experience with eCPR, a lack of training, unfamiliarity with workflows and ED environmental constraints (i.e., noise, lighting and equipment) [3, 7, 8]. 

This exploratory study focused on non-technical skills (NTS) in eCPR using pragmatic measures relevant to ED teams [8–10]. We adopted a working definition of NTS as “*cognitive*,* social*,* and personal resource skills that complement clinicians’ technical skills and contribute to safe and efficient task performance*.”^9^ We also drew on recent descriptions in the literature that outline approaches to measuring team performance using objective indicators (referred to by the authors as surgical sabermetrics). ^9–10^ In addition, Hicks’ work on human factors in the ED was used to implement both the design of the local eCPR system and the planning of the study [9–11]. 

The primary study aim was to observe OHCA responses and consider the potential role of self, team, environmental, and system factors on delivery of eCPR [9–14]. This focus addresses a relative lack of eCPR research that examines human factors [13–15]. The majority of eCPR studies focus on cost, efficacy or technical skills rather than the teams or the environmental challenges [13–17]. Consequently, little is known about human factors issues during eCPR cases, such as team coordination and communication [8]. Improved understanding of these factors could inform the design of eCPR systems and team training [14]. This exploratory study addresses this gap by observing non-technical aspects of eCPR delivery and drawing on perspectives from translational simulation, medical education, teamwork science and ergonomics research.

## Methods

### Study design

**Fig. 1:**
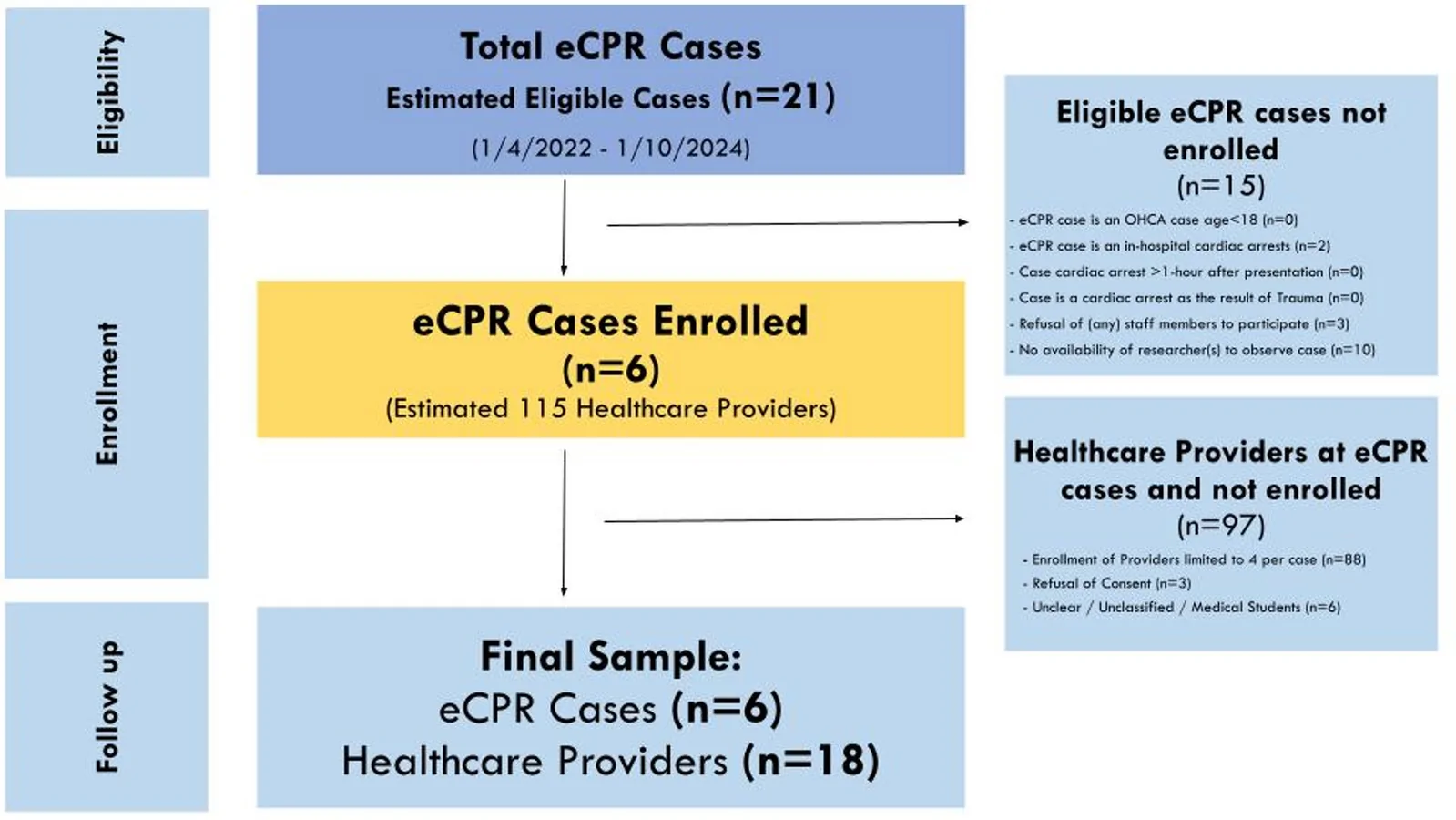
STROBE diagram for prospective observational study

A simulation-based preliminary phase (Phase 1) was followed by a longer exploratory observational phase during which eCPR cases were enrolled (Phase 2). In both phases, human-factors-related outcomes were the primary focus. The chosen approach was based on recent literature related to NTS, human factors, and resuscitation room design [9–12]. Key aspects are reported using a STROBE diagram (Fig. 1). eCPR cases did not receive intervention(s) outside of routine care following enrolment.

### Study setting

Ethics approval was granted prior to study commencement by the Western Sydney Local Health District Human Research Ethics Committee, Western Sydney Local Health District, NSW Health, Australia (Ref 2021/ETH11970). All study procedures were conducted in accordance with national standards, institutional research governance requirements, and the Declaration of Helsinki and its later amendments. The requirement for informed patient consent was waived by the approving ethics committee due to: (i) the impracticality of obtaining consent during cardiac arrest; (ii) the observational nature of the study; (iii) no change to clinical management; and (iv) the absence of identifiable patient data. Staff members enrolled in the study provided consent for participation. Written consent for publication was obtained from staff members shown in Fig. 3, which has also been reviewed and amended to ensure that no patient is identifiable. No identifiable patient information, images, or personal details are included in the manuscript.

Australian guidelines on handling of data and privacy were followed. The sites (Site 1: Westmead Hospital and Site 2: Royal Prince Alfred) are Level 1 Trauma and Cardiac Surgery centres. Both sites provide similar eCPR care, including highly selective ED-based eCPR for cardiac arrest cases [14]. 

### eCPR Models of Care

The eCPR response system is known as *“CODE ECMO”* at the two sites [14]. Teams were broadly similar in configuration, with pre-assigned key roles. Cannulation was typically performed by anaesthetic and intensive care medical officers, while leadership was provided by ED staff (Fig. 4). eCPR activations operate on weekdays between 0700 and 1700 with approximately one eCPR system activation per month at each site. This equated to approximately 2–4 cannulations per year at RPA and 5–7 per year at Westmead during the study period. The aligned approach to eCPR across the sites has been previously reported and the providers skill mix is also described in the paper by Oliver et al.^14^ CODE ECMO activations trigger both text and pager messages to pre-allocated eCPR providers (i.e. on-call pagers and a predefined messaging list). eCPR checklists are presented on a smartphone app available for various platforms (i.e., https://apps.apple.com/au/app/westmead-ecmo-app).

### Phase 1 (2-month *initial study phase*: 1st February 2022 to 31st March 2022)

The *initial study phase* (2 months) used simulation to map out a series of feasibility concerns related to observing actual eCPR cases. In this phase medical students (AS and PR) observed in-situ simulations and trauma cases with the aim of optimising the feasibility of observing eCPR (Phase 2). A notable component of the Phase 1 pilot was the deliberate use of simulation and trauma cases to refine data-collection processes before eCPR cases. This preparatory phase allowed testing of data sheets, anticipation of researcher workflow, and planning of participant follow-up. The value of simulation for refining research protocols is increasingly recognised. Benefits of using simulation in similar study designs have been outlined by Fatovich and co-authors who showed that embedding research into resuscitation simulation improved recruitment into complex clinical trials [15]. During this phase (Appendix 1) the investigators recorded individual, team, and environmental observations in real time. As a result, logistical challenges were identified, and an optimised data collection sheet was finalised for Phase 2 (clinical cases). The draft protocol included additional variables (video tracking, pedometers) but these were excluded after Phase 1 due to logistical concerns.

### Phase 2 (30-month *subsequent eCPR study phase*: 1st April 2022 to 1st October 2024)

eCPR cases were enrolled by convenience sampling. Enrolment relied on availability of investigators to observe (ARC, JC, AS, PR). Research assistants also contributed when available. The inclusion criteria were: (i) patient presenting with OHCA; AND (ii) activation of the eCPR team (criteria aligned with international standards); AND (iii) informed consent of directly observed eCPR team members before patient arrival. The exclusion criteria were: (i) age < 18; (ii) in-hospital eCPR (as cannulation typically occurs outside the ED, such as in the ICU or cardiac catheterisation suite, where workflow and environmental conditions differ and did not permit consistent measurement of study outcomes); (iii) cardiac arrest occurring more than 1 h after ED presentation (to preserve homogeneity); (iv) traumatic cardiac arrest; (v) refusal of provider(s) to participate; (vi) no availability of researcher(s) to observe. A majority of (provider) participants were consented in the resuscitation area during preparation time for the case arrival. A limited number (i.e., the 6 potential cannulators) were consented in advance to avoid interference with their real-time eCPR preparation due to consent.

### Sampling strategy

For a meaningful sample of “*observation of processes*,* team and health provider factors*” we aimed to enrol > 5 eCPR cases and > 20 discrete team members. No formal power calculation was performed. Team members such as eCPR cannulators routinely knew they were part of the team for any given day. Targeted key eCPR providers included Team Leaders (Medical, Crowd Control and Nursing), CPR (defibrillation) nurses, eCPR cannulators and airway doctors. For each case up to 4 team members were enrolled (i.e., capped by monitoring equipment and feasibility). eCPR team education encourages team members to be familiar with their role(s) and use team checklists (Fig. 4).

### Outcome measures

We selected outcome measures reflecting domains that may be relevant to eCPR performance. Multiple non-technical factors may be relevant to effective performance [8, 11]. Accordingly, we selected feasible, low-cost measures that matched Reid’s domains [18] (i.e., self, team, environmental and system). Observation of actual eCPR performance under real-world conditions yielded only a limited number of cases in which to examine these factors [8, 11]. We took the descriptions of data collection from work by Yule (sabermetrics), Hicks (trauma) and LeBlanc (resuscitation stress) as well as experiences from Phase 1 to select a final list of outcome measures [9, 14, 18]. 

The sampling approach sought to assess domains including: *Individual Factors (*focusing on individual response outcomes*); **Team Factors (*focusing on teamwork outcomes*) and ***Environmental Factors (*focusing on environmental outcomes*). The outcome measures used were conceptually linked rather than discrete and potentially prone to several biases that preclude causal inferences. In the discussion section, we provide (non-causal) interpretations of results drawing on literature related to non-technical skills and human factors in healthcare. This approach allows discussion of how these domains may shape performance. This informs a secondary goal of providing insights for eCPR training and systems. For each eCPR case, the two researchers aimed to collect the following data:

(A) Salivary Cortisol* *-* Hypothalamic-Pituitary-Adrenal system activation can be reliably assessed using salivary Enzyme-Linked Immunosorbent Assay (ELISA). Cortisol ELISA testing has narrow limits of agreement with plasma levels – peaks following stress occur within 1-hour [11, 19]. Participants were instructed to place a salivary swab in the mouth until saturated (approximately 1 min). Samples were placed in collection tubes and frozen. In this study samples were taken post-eCPR (a window 0.5 to 3 h after the case) and at ‘*baseline’* as a follow-up test (with a matched time to post-eCPR case sampling). Baseline samples were collected 2–3 weeks after the eCPR case during a comparable clinical shift and at a matched time of day to minimise diurnal variation. This approach led to a higher-than-expected rate of missing data due to failure of follow-up (Fig. 1).
(B) Heart Rate Monitoring* *-* Heart rate (HR) is considered a marker of physiological stress [20]. HR variability (HRV) is superior to raw HR but no suitable HRV devices were available. Polar H10 bands (Polar, Kempele, Finland) were used to measure continuous HR. These bands were linked to an iOS device (https://support.polar.com/au-en/polar-team-app*)* and monitored continuously with mean HR reported descriptively. Monitoring devices were applied during mustering phases before patient arrival with an aim to avoid interference with preparation. Baseline HR was not measured.
(C) State Trait Anxiety Index (STAI)* *-* The State-Trait Anxiety Inventory (STAI) is a widely used validated scale for measuring anxiety in the psychological literature. The STAI has two self-reporting scales (Y1-State and Y2-Trait). In a previous study of cardiac arrest simulations, the STAI was used to measure stress [11]. The self-reported items are summed to produce a total score. This can range from 20 to 80 with a high reported level of reliability [21]. The *state* scale was used to estimate post-event stress and consists of 20 statements (e.g., “I am tense”). Respondents rated their agreement with each statement on a four-point scale according to how they felt at that moment. STAI forms were used to collect *state* scores within 6 h of the eCPR event. STAI *trait* scores were also recorded at follow-up meetings with an investigator (e.g. when collecting baseline salivary cortisol tests). Reporting of STAI in this study is limited to descriptive elements due to the small sample size, a lack of validity evidence in comparing *state* and *trait* scores (Fig. 1).
(D) Teamwork Outcomes** - Mayo High Performance Teamwork Score (MHPTS) was measured in both phases of the study (16 items listed in Appendix 2). Additional measures of teamwork that were relevant to eCPR were also identified in the *initial study phase* (e.g. team leader summaries). These additional points of observation were included in the eCPR data collection form [14, 22, 23]. In the eCPR results we present the MHPTS score (from Appendix 2) supplemented by exploratory, non-validated observational descriptors. These supplementary elements were considered relevant to eCPR delivery and were based on work by Weller in New Zealand (Appendix 3) [23]. Two investigators recorded MHPTS scores, documented elements derived from Weller’s work and produced teamwork notes. Inter-rater reliability for MHPTS scoring was estimated using Cohen’s kappa (κ = 0.84), calculated at the item level across domains to quantify agreement between raters; we acknowledge this represents an approximation given the ordinal nature of the scale. In practice, the two observers were co-located during observations, which may introduce shared biases not captured by these measures. Any discrepancies in scoring were resolved by using the median of the two observers’ ratings for analysis.
(E) Environmental Outcomes*** - Ambient noise level (decibels, dB) was measured to characterise the acoustic environment. This was continuously monitored using a National Institute for Occupational Safety and Health (NIOSH) sound level meter with a calibrated microphone. The on-screen value was recorded at one-minute intervals (Appendix 3) [22] Noise levels have been measured extensively in operating rooms with similar methods [23–26]. A-weighted (current) levels (*LAeq*) and the C-weighted (peak) sound pressure (*LCpeak*) were displayed and recorded (Appendix 3). Microphones were placed on the left side of the resuscitation room, which formed part of an open space measuring approximately 55 m² (Fig. 3). Observations also included use of environmental controls (e.g. physical cordons such as retractable tape); positioning of eCPR team members in the space, and periods of ‘cross-talk’, defined as inaudible or overlapping episodes of communication.
(F) ***eCPR Case Outcomes*** Utstein outcomes were recorded for each case using a pre-existing OHCA registry [3]. Enrolment did not alter management. Cardiac arrest outcomes are reported that are relevant to appropriateness of eCPR selection (i.e. rhythm, time of arrest pre-hospital), the eCPR process (i.e., cannulation start time, finish time) as well as mortality at 24 h and 28 days.

### Analysis plan and bias

Analysis was conducted using IBM SPSS (version 19). Comparative analysis of the data was limited due to concerns about validity of comparative testing as well as the final sample size (Fig. 1). As a result, we describe most data descriptively. Comparative analysis of salivary cortisol used the Wilcoxon signed-rank test of available paired samples after a Shapiro–Wilk test suggested a non-parametric distribution. Attempts to control for confounders were limited but included matched timing of baseline and post-case cortisol testing.

Teamwork was assessed using the Mayo High Performance Teamwork Scale (MHPTS), which conceptualises observable behaviours associated with effective team performance. While the MHPTS describes a framework for describing ideal team behaviours, we recognise that observer-based scoring is inherently subjective. Observers used a standardised scoring approach informed by prior published studies, and inter-rater reliability was tracked but validity remains uncertain. Reid’s human factors framework was used as an interpretive lens to organise observations across individual, team, environmental, and system domains, rather than as a tool to generate causal inferences. Consistent with this approach, the study was designed to characterise patterns of team behaviour and generate hypotheses, rather than to draw definitive conclusions regarding eCPR team performance.

## Results

Table 1 outlines characteristics of the participants enrolled by convenience sampling over a 2-year period. This period commenced after a pilot (Appendix 1) that enrolled 13 cases. Data were collected from eight trauma ED cases, four trauma simulations and one eCPR simulation (Appendix 1). For the eCPR phase, 6 cases were enrolled with 5 from a single site. These cases provided 155 min of observable teamwork performance and enrolled 18 providers. Table 2 summarises the Utstein outcomes related to eCPR cases. Teamwork and ambient noise data are reported descriptively in this Table [14] Cases met local criteria for eCPR activation according to local guidelines, and 5/6 patients had a presenting shockable rhythm. Two patients were noted to have a period of return of spontaneous circulation (ROSC) [14]. There were no reported cannulation failures. Case 2 underwent cannulation but was *not* commenced on ECMO flow and was subsequently palliated. In this case, relevant clinical information led to the patient being palliated. Three patients were alive at 24 h of which two survived to hospital discharge. Missing data included: (i) dB Noise Levels (21 ‘*minutely*’ values); (ii) STAI Forms (8 providers did not complete); (iii) Cortisol Swabs (16 incomplete); (iv) Heart Rate (8 providers not monitored); (v) Headcount (7 ‘*count*’ values not recorded).

### Individual factors and stress

Among healthcare provider participants there were equal numbers of males and females. Most had a medical role (*n* = 11). Missing data related to these individuals are outlined in Fig. 1. Reports for heart rate, STAI and salivary cortisol are presented descriptively in Table 1. Mean heart rate was 97.0 bpm (SD 10.8). The mean baseline STAI trait score was 35.5 (SD 8.0) and the post-eCPR STAI (state) mean score was 41.8 (SD 9.67). Nine paired cortisol samples were available for analysis, with two samples excluded due to incomplete pairing. Median baseline cortisol was 2.0 nmol/L (IQR 3.0), compared with a post-eCPR median of 10.7 nmol/L (IQR 10.4; Wilcoxon signed-rank test, *p* = 0.009). Baseline samples were not obtained prospectively or with control for all potential confounders.

### Teamwork

Teamwork scores were consistently in a moderate to high range (*median MHPTS 12*,* IQR 10–13*). Suboptimal team behaviours included gaps in frequency of team summaries, attention not consistently drawn to ‘safety threats’, and a lack of consistency in closed loop communication. Headcount was observed in all eCPR cases and the crowded eCPR attendances were all higher than 18 providers (*median headcount 20; IQR 18–29*). Teamwork observations suggested that team leader summaries occurred more than once in most cases and were typically used to align the team to a shared understanding, rather than to support reflexivity or explicit decision-making. Despite high MHPTS scores, notable communication challenges were observed. Periods of loud, often inaudible, cross-talk occurred in four of six cases (typically ≥ 3 episodes per case).

Leadership communication behaviours were variable overall. While team leader summaries and explicit verbal instructions were observed intermittently, there was limited documentation of proactive attention to potentially hazardous actions or omissions. In four cases, leaders employed strategies to manage communication, including time-outs and visual cues (e.g. use of a “quiet” sign).

### Environmental factors and noise

**Fig. 2:**
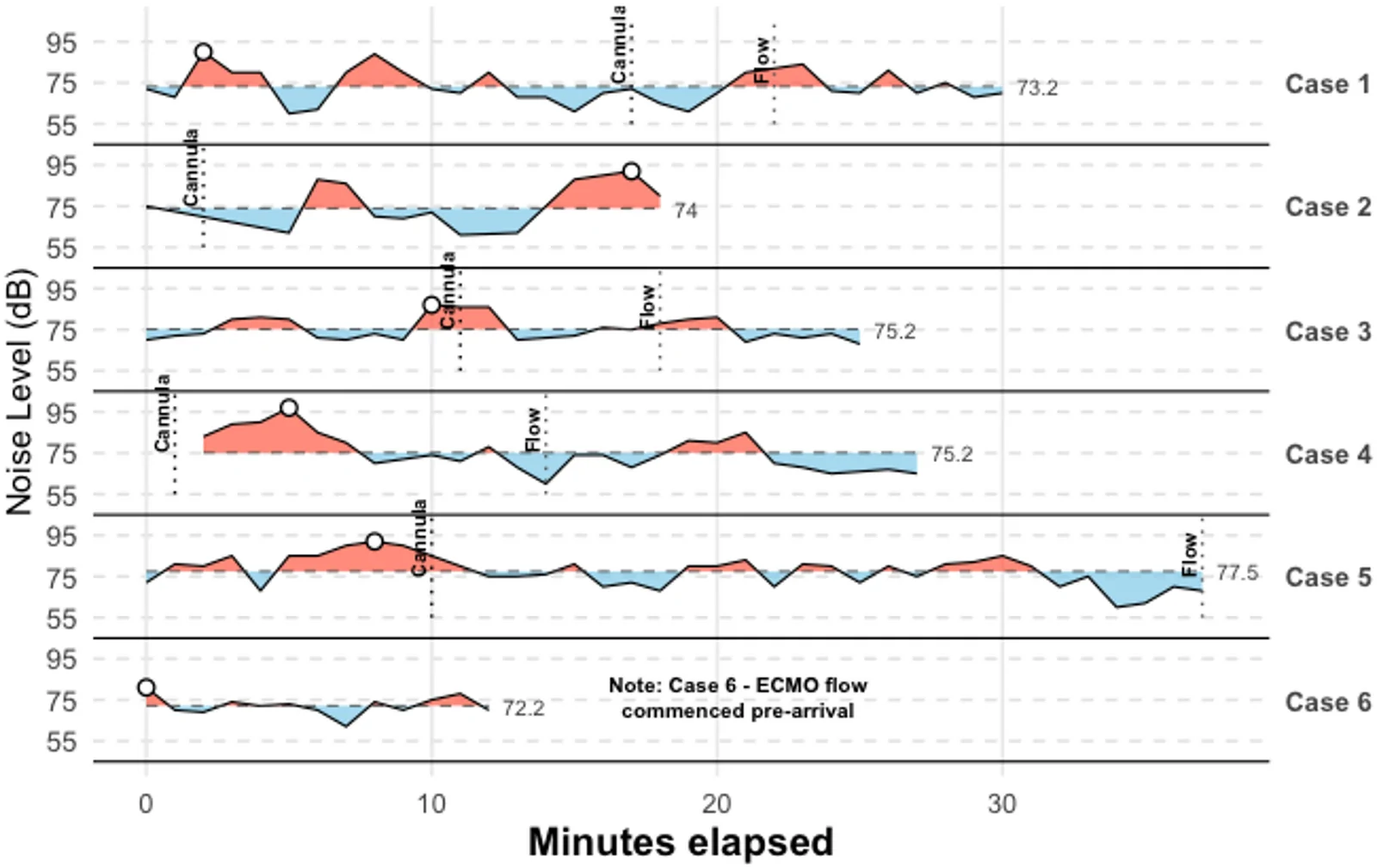
Continuous ambient noise level measurement in eCPR cases in the emergency department. Ambient noise in dB plotted each minute across six eCPR cases shown as a black line. Red and blue colours indicate when noise levels were above and below the case mean noise level respectively. Case mean noise levels are inscribed to the right of each plot. Peak noise levels are indicated by hollow points. Time points of successful cannulation and commencement of ECMO flow are labelled “Cannula” and “Flow” respectively

**Fig. 3:**
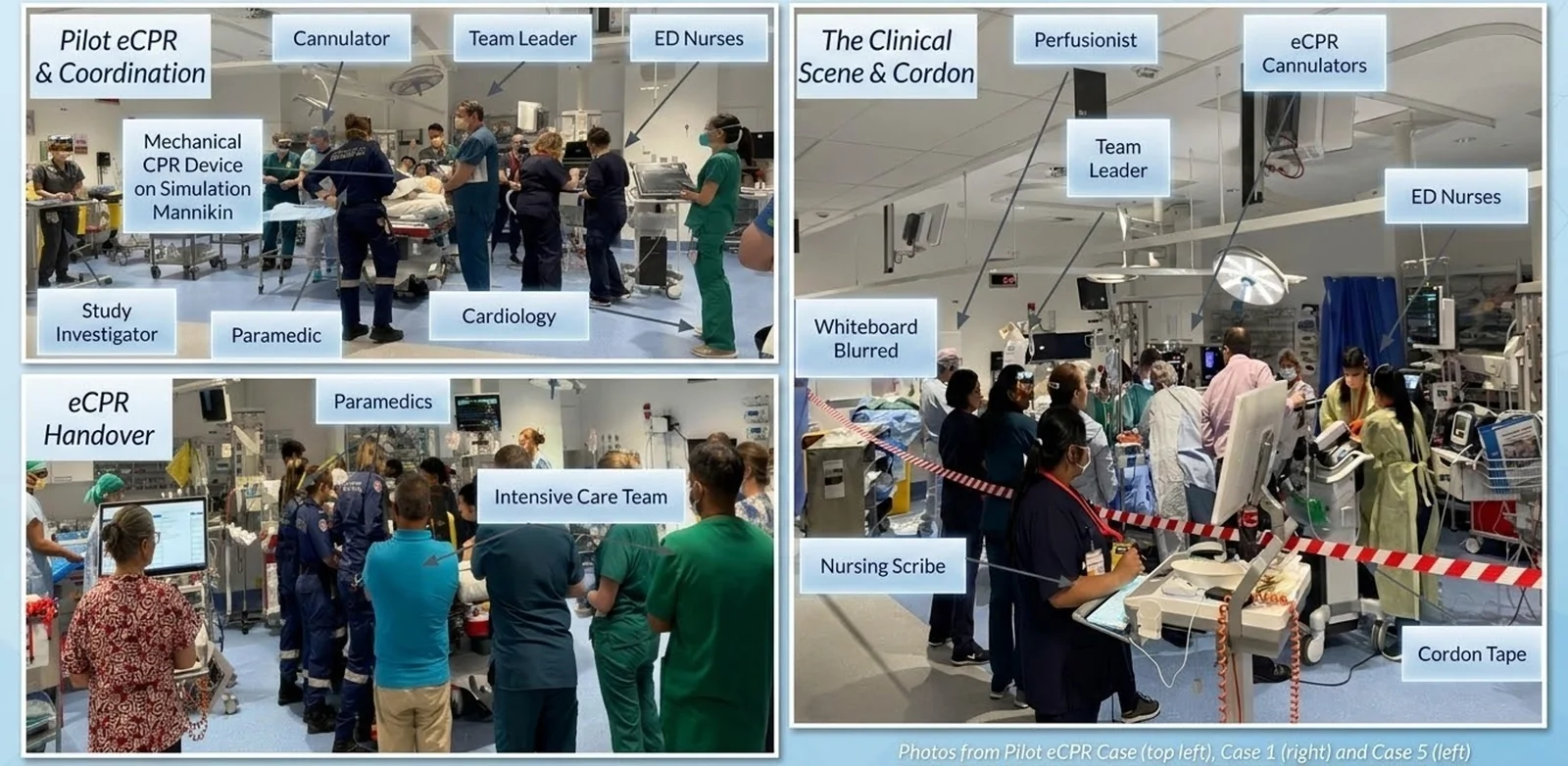
Visual representations from videos of eCPR cases (with permission from all participants)

Ambient noise data was collected for all eCPR cases in the same location. Baseline noise (without a patient) in the resuscitation area was measured over 10 iterations at 64.8 dB (SD 9.4) during the *initial study phase*. Noise levels during eCPR cases demonstrated a mean peak of 91.8 dB. The overall mean noise level was 74.6 dB (SD 4.69), representing the mean of all recorded time-point measurements across cases (not case-level averages) and presented descriptively due to non-independence within cases [23–29]. Fig. 2 illustrates the noise level over time for each eCPR case with cannulation timings superimposed. A physical cordon (Fig. 3) separating clinical and corridor space was used in three cases. Typically, most eCPR attendees remained outside of the clinical space regardless of the use of a cordon. For example, in Case 1 many providers (*n* = 32) attended but only 11 team members (Fig. 3) were observed inside the designated resuscitation area.

**Table 1:** Description of eCPR team healthcare providers

Team Members, Sites and Case Number	eCPR Role	Heart Rate mean (SD)	Experience (post-graduate years)	*STAI (Y1 STATE) (1–3 h post eCPR)	Baseline Salivary Cortisol (nmol/L)	Cortisol 1–3 h post eCPR (nmol/L)	*STAI (Y2 TRAIT) (Recorded at Baseline)
Provider 1 (Case 1 / Site 1)	Medical Team Leader	109.8 (6.8)	7	Not Recorded	Not Recorded	Not Recorded	Not Recorded
Provider 2 (Case 1 / Site 1)	Defibrillation Nurse	73.0 (8.2)	13	Not Recorded	Not Recorded	Not Recorded	Not Recorded
Provider 3 (Case 1 / Site 1)	Anaesthetist Cannulator	86.6 (17.3)	15	46	Not Recorded	Not Recorded	46
Provider 4 (Case 2 / Site 1)	Medical Team Leader	101.2 (7.4)	19	43	3.6	9.5	34
Provider 5 (Case 2 / Site 1)	Crowd Control Leader	Not Recorded	17	45	2.1	12.9	32
Provider 6 (Case 2 / Site 1)	Anaesthetist Cannulator	104.8 (14.8)	26	21	2.0	6.1	24
Provider 7 (Case 2 / Site 1)	Defibrillation Nurse	Not Recorded	7	35	0.6	2.7	35
Provider 8 (Case 3 / Site 2)	Defibrillation Nurse	Not Recorded	8	56	6.7	16.7	30
Provider 9 (Case 3 / Site 2)	Medical Team Leader	91.5 (10.2)	27	Not Recorded	1.9	8.1	Not Recorded
Provider 10 (Case 3 / Site 2)	Theatre Scout Nurse	103.8 (11.1)	7	50	0.6	2.7	52
Provider 11 (Case 3 / Site 2)	Crowd Control Leader	Not Recorded	13	46	6.6	14.1	37
Provider 12 (Case 4 / Site 1)	Anaesthetist Cannulator	Not Recorded	19	Not Recorded	Not Recorded	Not Recorded	Not Recorded
Provider 13 (Case 4 / Site 1)	Defibrillation Nurse	Not Recorded	30	Not Recorded	0.6	2.9	Not Recorded
Provider 14 (Case 5 / Site 1)	Medical Team Leader	98.7 (10.9)	21	Not Recorded	2.1	Not Recorded	Not Recorded
Provider 15 (Case 5 / Site 1)	Defibrillation Nurse	93.3 (3.5)	28	Not Recorded	Not Recorded	Not Recorded	Not Recorded
Provider 16 (Case 6 / Site 1)	Medical Team Leader	Not Recorded	20	41	1.1	Not Recorded	32
Provider 17 (Case 6 / Site 1)	Responding Anaesthetist	Not Recorded	15	Not Recorded	Not Recorded	Not Recorded	Not Recorded
Provider 18 (Case 6 / Site 1)	Defibrillation Nurse	107 (9.3)	6	35	Not Recorded	Not Recorded	33

*STAI results are presented in this table (Trait and State scales) but have not been shown to be valid as comparative scores

**Table 2:** Description of eCPR cases and teamwork factors

	Variable	Case 1	Case 2	Case 3	Case 4	Case 5	Case 6
Demographic and Utstein Outcomes	Age (decade/range)	40–49	50–59	50–59	30–39	30–39	50–59
	Location	Site 1	Site 1	Site 2	Site 1	Site 1	Site 1
	Presenting Rhythm	VF	VT/Torsades	VF	VF	PEA	VF
	Pre-hospital Time (mins)	50	32	50	66	38	(N/A)
	Total Time in ED (mins)	31	19	27	38	28	12
	ROSC in ED (pulse returned during time in ED)	No	Yes	No	No	No	Yes^
	eCPR Cannulation Success (in correct locations)	Yes	Yes	Yes	Yes	Yes	Yes
	eCPR (on pump) flow in ED	Yes	No **†**	Yes	Yes	Yes	Yes*
	24-hour mortality	Died	Died	Alive	Died	Alive	Alive
	28-day mortality	Died	Died	Alive	Died	Alive	Died
	Noise (dB; case mean) [range of readings] (peak recorded reading)	73.2 dB [60–96] (Peak 96 dB)	74.0 dB [61–94] (Peak 94 dB)	75.2 dB [68–91] (Peak 91 dB)	75.2 dB [60–96] (Peak 96 dB)	77.5 dB [60–94] (Peak 94 dB)	72.2 dB [62–80] (Peak 80 dB)
Team Outcomes	Mayo High Performance Teamwork Score (Range of Scores 0–16)	12	10	13	12	10	14
	Highest Provider Headcount at any time during eCPR case	Inside 11 Outside 21 (Total 32)	Inside 9 Outside 9 (Total 18)	Inside 10 Outside 16 (Total 29)	Inside 12 Outside 8 (Total 20)	Inside 10 Outside 8 (Total 18)	Inside 14 Outside 10 (Total 24)
	Provider Headcount at eCPR flow start	Inside 18 Outside 10	N/A	Inside 9 Outside 20	Inside 6 Outside 12	Inside 4 Outside 9	Inside 12 Outside 8

**†-**Sheaths and eCPR Cannulas inserted but palliated (no ECMO flow); **^**-Pulsatile Flow on Arterial Line; *-Pre-hospital eCPR case (arriving with cannulas)

## Discussion

Cardiac arrest management in the ED setting is inherently time-pressured and stressful, with the additional demands introduced by escalation to eCPR delivery requiring effective teamwork [30]. Within the limitations of this small exploratory dataset, several descriptive patterns were observed. For example, teamwork scores were in the moderate to high range, events were stressful at an individual level, and the resuscitation spaces were both crowded and noisy. These observations are familiar features of high-acuity care in the ED setting.

Given the limitations of the exploratory study design, we deliberately avoid overstating generalisability of outcomes or making substantive causal claims. In this discussion the data are interpreted in relation to existing literature and clinical context. Team coordination and systems could affect performance during high-acuity, low-occurrence (HALO) events [18]. Therefore, we also used Reid’s four domains as an analytical lens and overarching structure for the discussion. The domains are: (i) *individual* factors; (ii) *teamwork* issues; (iii) *environmental* influences; and (iv) *system* considerations [8, 18]. 

### Individual factors and stress

Salivary cortisol, interpreted cautiously, was compared between baseline and post-eCPR and was higher post-eCPR compared with baseline (Wilcoxon signed-rank test, *p* = 0.009). Observed increases in cortisol (mean > 6 nmol/L) are similar to ranges reported in other studies [11, 31, 32]. Heart rate and psychological data are interpreted with caution as no statistical comparisons were made [21]. Results from larger studies demonstrate increased individual stress responses [11, 33–35]. For example, a randomised study by Lizotte and co-authors examined simulated neonatal emergencies. In cases of cardiac arrest, the investigators observed significant stress in participants [33]. Despite a measurable increase in stress, the teamwork remained consistently high in keeping with the limited observations from this study.

Stress is widely known to influence healthcare provider performance [35–37]. If one considers stress as a continuum, low levels of arousal and unattenuated hyperarousal can both lead to underperformance [8]. The limited data available that relates to HALO events suggests unmanaged stress inhibits performance [8, 35]. One study previously reported that stress leads to attenuated perceptions, limited ability to perform tasks and poor allocation of attention [30]. The concept of ‘*Stress Inoculation Training (SIT)*’ has been cited as one strategy that aids future performance when faced with stressful situations. Proponents of SIT suggest that stress is *‘inevitable’* in many healthcare environments, including EDs [37–39]. The effectiveness of this training has not yet been clearly demonstrated. However, SIT is one factor, that was present at the two study sites and may represent a contextual factor [40, 41]. 

Observations of stress during eCPR align with work by Folkman and Lazarus [42]. Their work, which underpins modern conceptualisations of stress, suggests that performance may be determined by perception of the *‘level of demand’* versus the ‘*resources available*’ to manage a situation [42]. For example, if healthcare providers perceive that resources are not sufficient to match demand then there is a risk of inhibiting performance. Conversely, balanced perception leads to “*reframing*” the situation to a challenge rather than a threat [8]. These ideas could be a focused target of training for eCPR events [43]. The literature reports that the effectiveness of stress training education programmes may depend on skilled pre-briefing and debriefing. Evidence suggests that calibration of the level of stress and management of anxiety among learners is also important [38, 40, 44]. Therefore, if planning eCPR training we should prioritise skilled facilitation and avoidance of ‘over-stressing’ the learners [40, 44]. 

### Teamwork issues

Teamwork was assessed using a validated instrument supplemented by study-specific observational descriptors (Fig. 1) [14, 22, 23]. While the use of clinician raters introduces a potential risk of rater bias, inter-rater agreement was reasonable, indicating acceptable consistency of scoring. Teamwork scores during eCPR were rated in the moderate to high range. It should be acknowledged that numerical scores may not fully capture the lived experience of teams; these findings should therefore be interpreted as descriptive rather than definitive indicators. When comparing these findings to other studies using MHPTS, the results were similar. These studies showed that pre-briefing, self-appraisal and mental rehearsal often led to effective teamwork.

Prior work has demonstrated that pre-briefing, self-appraisal, and mental rehearsal support effective teamwork, particularly in high-acuity settings and most studies have reported high ratings of teamwork [45–47]. Sørensen and colleagues have highlighted high-quality communication, non-technical skills training, and shared mental models as key performance safeguards for teams [30, 48]. Several of these elements were noted in eCPR cases; for example, team leaders frequently provided structured summaries (Table 2). Other studies have suggested effective teams often display elements of adaptability and an ability to manage uncertainty [49, 50]. One potential target for teamwork training identified for future work was the creation of opportunities for team members to speak up. Effective speaking-up behaviours are contingent on environmental and systems factors, including audibility and turn-taking. In addition, the regular occurrences of ‘*cross-talk’* (Table 2) and high levels of ambient noise are potential targets for local education and specific research. The wider literature highlights the importance of ongoing attention to teamwork training in complex healthcare environments.^43 48^

### Environmental factors and noise

Noise during eCPR events may include various sources including team communication and device operation, such as mechanical CPR machines. ^25–28^ There appeared to be case noise variation, with marked peaks and troughs occurring around procedural phases such as cannulation and initiation of ECMO flow. Peak levels were comparable to those reported by Short et al., though ambient noise levels we observed were higher than the baseline levels described in this work [51]. Evidence from operating theatre settings suggests that noise is associated with teamwork processes and may reflect periods of task demand. Measured noise has been described as an indicator of task intensity [52]. Noise ‘peaks’ may reflect both constructive behaviours, such as task-relevant communication (i.e. team coordination) and represent task-irrelevant noise [27–29, 53]. A recent review of 16 operating room studies reported mean noise levels ranging from 51 dB to 79 dB.^28^ The World Health Organization recommends substantially lower noise levels in clinical environments than those observed.^28 51^ Australian workplace standards advise that sustained exposure above 85 dB should prompt consideration of mitigation strategies. Work from North American operating room “black box” studies by Grantcharov and colleagues supports the use of multiple objective measures, including noise, to characterise performance [54]. Applying similar approaches to eCPR teams appears feasible from this exploratory work. Future work could explore the use of such metrics for research, feedback, and quality improvement. Noise may also represent an observable marker of task intensity [55].

### Systems, workflow and crowd control

**Fig. 4:**
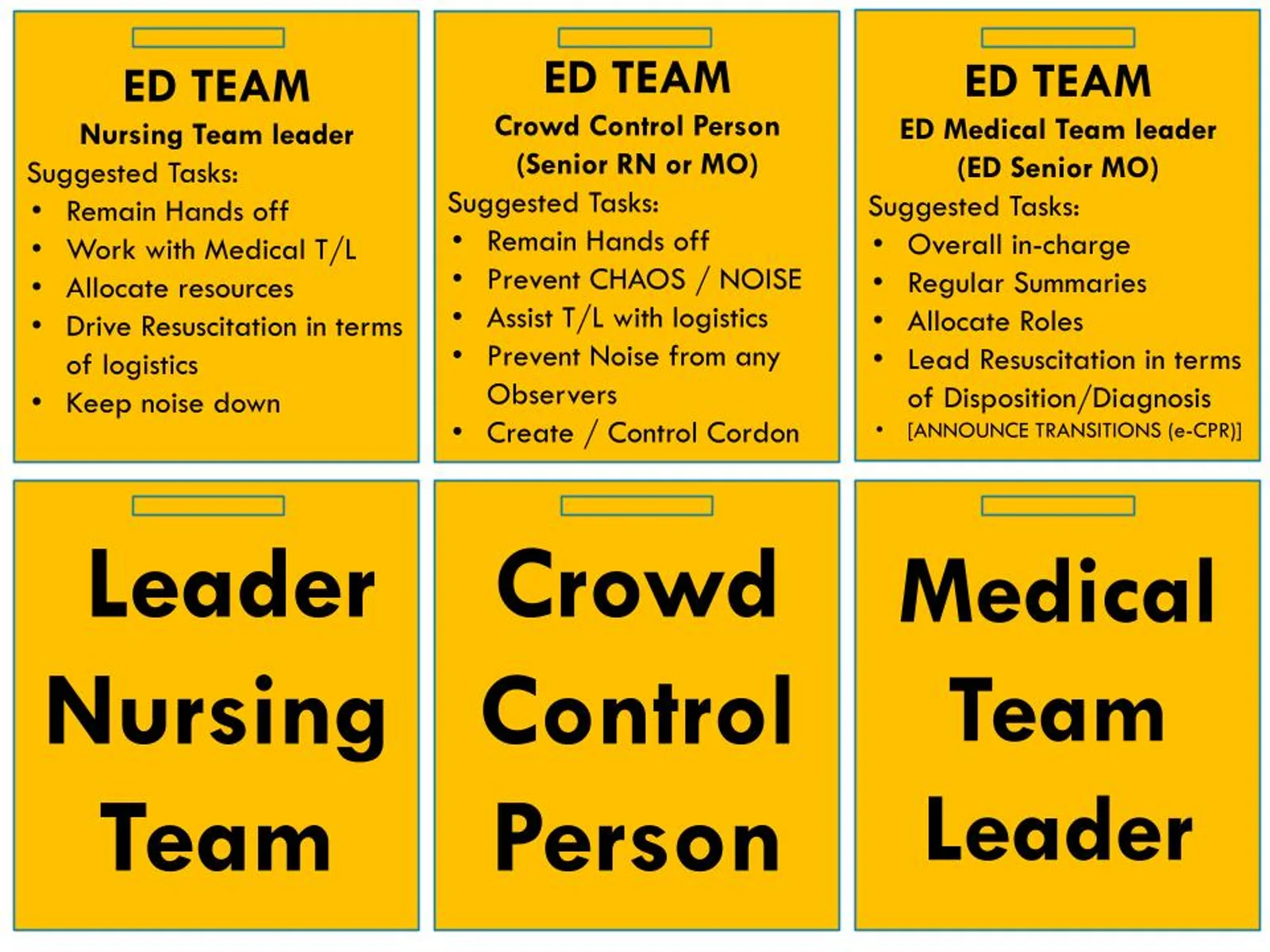
Lanyard role cards for “3 Leader” roles during eCPR

The last domain for consideration is systems [8]. Illustrations of the eCPR setup are shown in Fig. 3, with design details described previously [14]. In refining the eCPR system, in situ simulation and tabletop exercises were used to map workflow. The use of simulation aligns with ED systems work described by Hicks and emerging literature on Visually Enhanced Mental Simulation (VEMS) [8, 56, 57]. Changes arising from these exercises included ergonomic positioning of ultrasound devices, pausing defibrillation during cannulation, introduction of a lanyard-based role allocation (Fig. 4), and use of a tape cordon (Fig. 3) to limit crowding by observers [14]. 

One notable system design feature was an introduction of crowd control leaders (CCLs). This role was allocated to a senior provider (Fig. 4) [49]. Relational coordination refers to timely, accurate, and problem-solving communication supported by shared goals and mutual respect, enabling effective teamwork [49]. A CCL could support relational coordination by avoiding engagement in clinical tasks and supporting other team members (Table 2) [8, 44, 58]. 

### Limitations

Several significant limitations warrant further consideration. The small sample size (six eCPR cases involving 18 healthcare providers) limits generalisability. All but one eCPR case was observed at a single site, limiting the ability to support meaningful multi-site comparison or inference. In addition, several eligible eCPR cases were not captured due to logistical constraints, introducing potential selection bias. Observational bias, including a possible Hawthorne effect, may also have influenced results, although this was mitigated through prospective consent and positioning observers in less conspicuous locations. Missing data were substantial, with physiological stress measures available for only around half of participants, further limiting interpretability. Finally, the absence of blinding, control groups, and longitudinal follow-up precludes definitive conclusions being drawn from these data.

A strength of this study was the structured use of simulation and non-eCPR cases to iteratively refine data collection prior to observing live cases. The use of a recognised conceptual framework also provided theoretical grounding for interpretation.

## Conclusions

This exploratory study describes the interaction of individual, team, environmental, and system factors during real-world eCPR events. Across a small number of eCPR cases, we observed individual stress, noise levels, and large multidisciplinary teams operating in crowded spaces. Observable teamwork assessed using a structured NTS framework was maintained, although limitations in data quality preclude causal inference. Unproven system-level features such as role allocation were also observed. This limited exploratory study provides preliminary hypothesis-generating insights for supporting teamwork in eCPR cases and a foundation for further observational or ethnographic research exploring stress, teamwork, and environmental issues related to resuscitation.

## Supplementary Information

Below is the link to the electronic supplementary material.

Supplementary Material 1

Supplementary Material 2

Supplementary Material 3

## Data Availability

All data relevant to the study are included in the article or uploaded as supplementary information.

## Abbreviations

CCL: Crowd control leader
CPR: Cardiopulmonary resuscitation
ED: Emergency department
eCPR: Extracorporeal cardiopulmonary resuscitation
ECMO: Extracorporeal membrane oxygenation
HALO: High-acuity, low-occurrence
HR: Heart rate
MHPTS: Mayo High Performance Teamwork Scale
NTS: Non-technical skills
OHCA: Out-of-hospital cardiac arrest
ROSC: Return of spontaneous circulation
STAI: State-Trait Anxiety Inventory
