# Supplementary material for: Emergency department eCPR: an exploratory observational study of human factors
Source: BMC Emerg Med. 2026 Jun 17;26:233. doi: 10.1186/s12873-026-01650-4 (PMC13520427; doi:10.1186/s12873-026-01650-4)
Supplement: Supplementary file 2 [file 12873_2026_1650_MOESM2_ESM.docx]

**Appendix 2 – Modified Mayo High Performance Teamwork Scale (MHPTS)**

Adapted from - Malec JF, Torsher LC, Dunn WF, Wiegmann DA, Arnold JJ, Brown DA, Phatak V.The mayo high performance teamwork scale: reliability and validity for evaluating key crew resource management skills. Simul Healthc. 2007 Spring;2(1):4-10.

**MHPTS TEAM CHECKLIST**

Use the following scale to rate the team on each dimension:

0	1	2

Never or rarely	Inconsistently	Consistently

Please rate conservatively. Most teams that have not worked extensively together do not consistently demonstrate many of the qualities described in the scale.

**Always rate items 1-8. (MAIN SCORE UP TO 16)**

(1) A leader is clearly recognised by all team members.

______________________________________________

(2) The team leader assures maintenance of an appropriate balance between command authority and team member participation without a loss of situational awareness.

______________________________________________

(3) Each team member demonstrates a clear understanding of his or her role.

______________________________________________

(4) The Team Prompts each other to attend to significant clinical indicators throughout the procedure/intervention.

______________________________________________

(5) When Team Members are actively involved with the patient they verbalise their activities aloud.

______________________________________________

(6) Team members repeat back or paraphrase instructions and or seeking clarification to indicate that they heard them correctly.

______________________________________________

(7) Team members refer to established protocols and checklists for the procedure/intervention.

______________________________________________

(8) All members of the team are appropriately involved and participate in the activity.

______________________________________________

TOTAL SCORE (0-16) ______________________________

Items 9-16 may be marked or "NA (not applicable)"

Scored as above and/or make **FIELD NOTES**

(9) Disagreements or conflicts among team members are addressed.

______________________________________________

(10) When appropriate, roles are shifted to address urgent or emergent events.

______________________________________________

(11) When directions are unclear, team members acknowledge their lack of understanding and ask for repetition and clarification.

______________________________________________

(12) Team members acknowledge — in a positive manner — statements directed at avoiding or containing errors or seeking clarification.

______________________________________________

(13) Team members call attention to actions that they feel could cause errors or complications.

______________________________________________

(14) Team members respond to potential errors or complications with procedures that avoid the error or complication.

______________________________________________

(15) When statements directed at avoiding or containing errors or complications do not elicit a response to avoid or contain the error, team members persist in seeking a response.

______________________________________________

(16) Team members ask each other for assistance prior to or during periods of task overload.

____________________________________________________
