# Supplementary material for: Emergency department eCPR: an exploratory observational study of human factors
Source: BMC Emerg Med. 2026 Jun 17;26:233. doi: 10.1186/s12873-026-01650-4 (PMC13520427; doi:10.1186/s12873-026-01650-4)
Supplement: Supplementary file 3 [file 12873_2026_1650_MOESM1_ESM.docx]

**Appendix 1 - Pilot Study Dataset**
